# Advance care planning and goals of care discussion: the perspectives of Brazilian oncologists

**DOI:** 10.1186/s12904-022-01052-w

**Published:** 2022-09-22

**Authors:** Laiane Moraes Dias, Mirella Rebello Bezerra, Williams Fernandes Barra, Ana Emília Vita Carvalho, Luísa Castro, Francisca Rego

**Affiliations:** 1 Faculty of Medicine of the University of Porto, Porto, Portugal; 2 João de Barros Barreto University Hospital, Federal University of Pará, Dom Romualdo de Seixas, 1476/2207, Belém, PA 66055-200 Brazil; 3 IMIP, Instituto de Medicina Integral Professor Fernando Figueira, Recife, PE Brazil; 4 CESUPA, Pará University Center, Belém, PA Brazil

**Keywords:** Personal autonomy, Oncology, Advance care planning

## Abstract

**Background:**

Advance care planning (ACP) and goals of care discussions are important instruments that enable respect for patient autonomy, especially in patients with a life-threatening disease, such as cancer. Despite their well-established benefits, ACP and goals of care discussions are still not frequently performed in clinical oncology practice. Understanding the barriers to this topic is the first step toward developing future interventions that are more likely to improve professional practice and patient satisfaction with care.

**Aim:**

To explore Brazilian oncologists’ barriers to discuss goals of care and advance care planning.

**Methods:**

A cross-sectional study was developed to identify Brazilian oncologists’ barriers to discussing goals of care and ACP. The *Decide-Oncology* questionnaire was used to identify the importance of these barriers according to oncologists’ perceptions. Participants were asked to rank the importance of various barriers to discussing goals of care, ranging from 1 (extremely unimportant) to 7 (extremely important). A quantitative analysis using descriptive statistics was used, including median and interquartile intervals and a qualitative analysis based on Bardin content analysis of the two open questions.

**Results:**

Sixty-six oncologists participated in this study. Most of them perceived the patient and family’s related barriers as the most important, such as patients’ difficulty in understanding their diagnosis and accepting their prognosis. Physician and external related factors, such as lack of training and lack of time for this conversation, were also described as important barriers. Participants with formal training regarding goals of care communication and with experience in palliative care perceived the lack of patients’ advanced directives as a significant barrier and manifested more willingness to participate in decision-making about goals of care. The lack of access and of support for referral to palliative care was also considered a significant barrier for ACP and goals of care discussion.

**Conclusion:**

The identification of barriers that limit the discussion of ACP and early palliative care referrals can certainly help to prioritise the next steps for future studies aimed at improving ACP and helping clinicians to better support patients through shared decision-making based on the patient’s values and experiences.

**Supplementary Information:**

The online version contains supplementary material available at 10.1186/s12904-022-01052-w.

## Introduction

Evidence shows that most patients want to discuss end of life plans with their doctors, despite this still being an uncommon practice [1]. Communicating about the end of life has been reported as one of the most difficult and stressful parts of the oncological practice, given that oncologists receive little training in this area. As a result, many do not communicate these issues effectively with their patients [2]. Many patients with advanced cancer spend their last month of life in the hospital, with 6% percent receiving chemotherapy up to 2 weeks before death [3].

One of the principles of good care is respect for patients’ wishes and values; thus, it is important to understand patients’ perspectives about cancer treatment [4]. Furthermore, evidence suggests that unwanted treatment at the end of life is associated with negative outcomes, such as reduced quality of life and low satisfaction with care [4, 5].

In this context, advance care planning (ACP) and goals of care discussion are justified as instruments that enable respect for the patient’s autonomy [6]. They involve the exploration of a person’s values, beliefs, and what is most important to each person: to ensure concordance between the received clinical care and the patient’s wishes [5, 7]. It is important to note that ACP conversations focus on preparing for future healthcare decisions, whereas goals of care discussion focus on current healthcare decisions. Thus, goals of care discussions are also an important part of the ACP process [5, 7, 8, 9, 10].

ACP is associated with a wide variety of benefits, such as less moral distress of health care professionals; higher rates of patient advanced directives (AD); reduced hospitalisation and intensive and futile treatment at the end of life; greater probability of the patient dying at the chosen place; greater satisfaction with the quality of care; and less risk of stress and depression in family members during bereavement [5, 11]. But even in the face of its benefits, ACP is still very little performed in clinical practice [9]. To know the barriers to goals of care discussion enables the development of tailored interventions that are more likely to improve professional practice and training programs [12, 13]. Considering the scarcity of studies about the difficulties of Brazilian oncologists in discussing ACP and goals of care, this study aims to identify these barriers.

## Aim

To explore Brazilian oncologists’ perceived barriers to discuss goals of care and advance care planning with the patient.

## Methods

### Type and questionnaire study

A cross-sectional study with a quantitative and qualitative approach was developed to identify Brazilian oncologists’ barriers to discussing goals of care and ACP. A sociodemographic questionnaire was included (age, gender, ethnicity, religion and years of experience in oncology). The instrument *“Decide-Oncology”* [14] was used to identify the importance of these barriers according to the perception of physicians who assist cancer patients. The *DECIDE (DECIsion-making about goals of care for hospitalized meDical patiEnts*) questionnaire was originally developed to identify barriers and facilitators to improve EOL communication and decision-making with critically ill hospitalized medical patients with advanced chronic illness. In 2017, Ethier et al. [14]. adapted and validated the DECIDE questionnaire to the oncology context, in a Canadian multicentric survey involving only oncologists. This instrument is based on three pillars: (1) barriers to the discussion about goals of care; (2) barriers to the approach of interrupting active cancer therapies (for example, chemotherapy, radiation therapy); and (3) barriers to referring to palliative care teams. These difficulties were further stratified into barriers related to patients, doctors, and/or related to the health system or external factors. Each session comprises statements about the difficulties in discussing advance care planning in a Likert-type response scale classified from 1 to 7 points, where 1 is the extremely unimportant difficulty; 2, very unimportant; 3, little unimportant; 4, neither important nor unimportant; 5, little important; 6, very important; and 7, extremely important [14].

Questions about participants’ degree of formal training on ACP and discussing goals of care and their perception of its importance, as well as participants’ suggestions to improve decision-making about goals of care in clinical practice, to better qualitatively evaluate such strategies, were comprised to meet a sequential explanatory design.

The *“Decide-Oncology”* questionnaire underwent a translation process to Portuguese—Brazil, followed by a cross-cultural adaptation by experts in the area to content validity and the translation's adequacy [15]. A Content Validity Coefficient > 0.80 was considered [15, 16]. Further details of the cross-cultural adaptation process are described in the pilot study already submitted for review for publication [10].

The questionnaire was adapted into an electronic version (Google forms) and sent via e-mail to the Brazilian oncologists via the Brazilian Society of Clinical Oncology.

### Study population

Sixty-six oncologists from all Brazilian regions responded to the questionnaire (out of approximately 700 Brazilian oncologists) after two rounds of electronic survey via e-mail with an interval of two weeks (39 answered in the first round and 27 in the second round).

### Statistical analysis

The results of the paper and electronic surveys were pooled and compiled using descriptive statistics, including median and interquartile intervals [1^st^ Q; 3^rd^ Q] for quantitative variables and counts and proportions for categorical variables. Survey responses were presented by median scores. The distribution of scores was compared between groups of participants who had formal training on communication about goals of care and those who had no training, as well as between groups who had already worked at palliative care services at some point and those who had not worked, using Mann–Whitney’s non-parametric test. Data analysis was performed using SPSS® Statistics (version 26.0; SPSS Inc., Chicago, IL, USA). In all tests, values of *p* < 0.05 were considered significant.

### Qualitative analysis

A content analysis based on Bardin [17] was conducted, and two categories, namely intrinsic and extrinsic factors, were developed based on the content found in the answers to the two open questions: *1- “Reflecting on the barriers which you rated as very important or extremely important in Sect. 1, what specific suggestions do you have about ways to overcome these barriers and make it easier for healthcare providers to talk with patients and their family members about goals of care?” 2- “What is currently working well to promote communication and decision-making about goals of care between healthcare providers and patients and their family members?”.*

As these questions were related to the unique issue “*Suggestions to improve communication and decision-making about goals of care*”, we condensed the answers according to the content analysis. A system of categories was developed to analyse the contents following three phases: pre-analysis (contact with the material to be analysed, detailed reading of the content); exploration of the material (choice of coding units, classification, grouping of words by meaning); and treatment of results – inference and interpretation (identification of the latent content, the meaning behind the apprehended content) [17]. This process was conducted by two authors independently, and they then reached a consensus about the categories.

## Results

### Quantitative analysis

#### Participants

Sixty-six oncologists from all Brazilian regions answered the electronic surveys between April and September 2021. Most participants were female (*n* = 38; 57.6%), with a median age of 40 years old (minimum of 28 years and maximum of 68 years of age). Considering the five regions of Brazil, most participants (*n* = 27;40.9%) act in the Southeast region, followed by the North region (*n* = 17;25.7%). The participants have a median of 11.5 years in oncology practice. Half of the participants have already worked in palliative care settings. Respondent demographics are outlined in Table 1.

A) Barriers to discussing ACP and goals of care

**Fig. 1:**
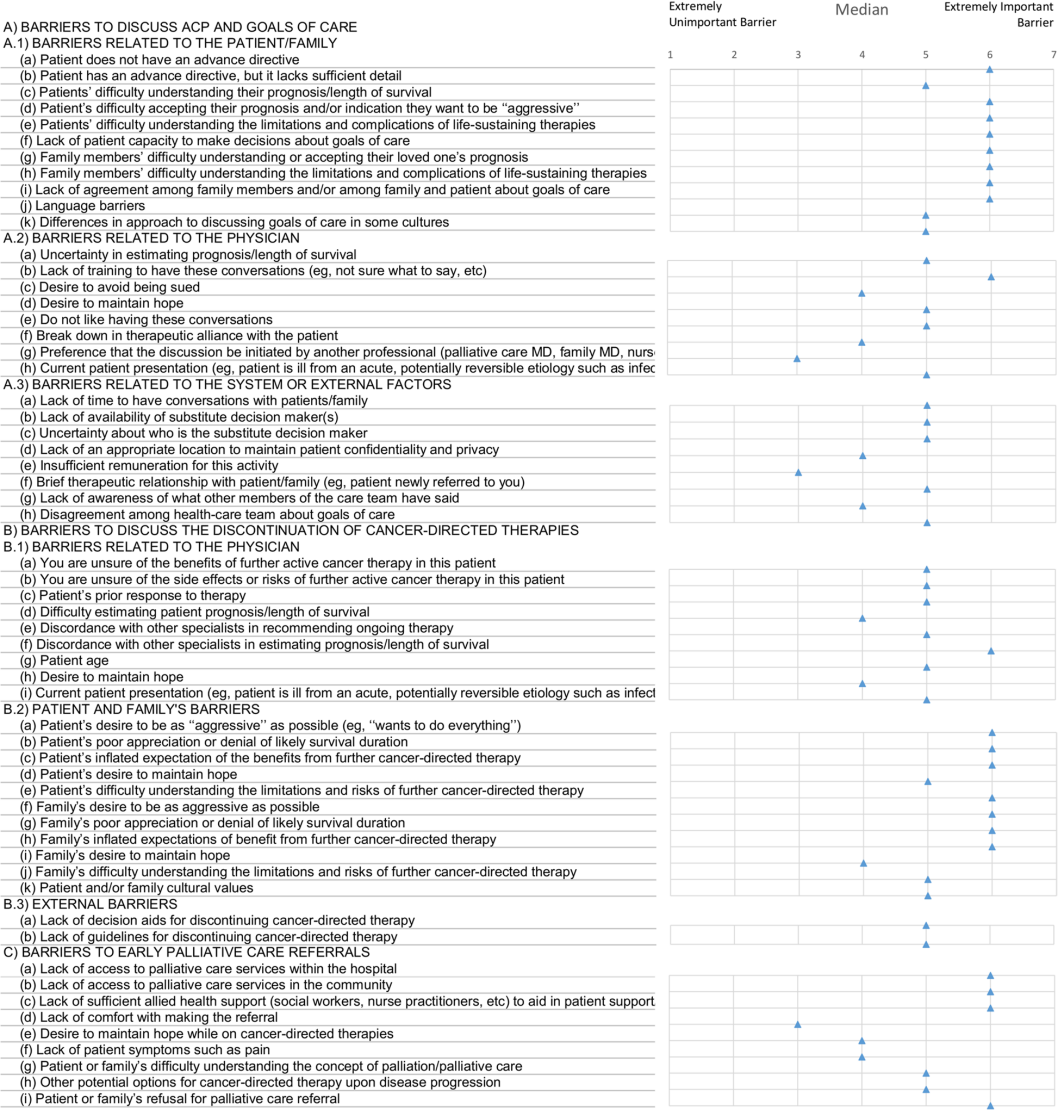
Barriers of patient, physician, and external factors to initiating goals of care discussions (**A**), interrupting cancer-directed therapies (**B**), and referring to palliative care (**C**) (median scores)

Most oncologists perceived the patient and family’s related barriers as the most important, such as patients’ difficulty in understanding their diagnosis and accepting their prognosis, patients’ desire to receive full active treatment, and lack of an advance directive (Fig. 1).

Uncertainty in estimating prognosis/length of survival and lack of training were the main physician-related barriers, despite most of the participants already having had formal training regarding communication. And the main external factor indicated was a lack of time to have a conversation (Fig. 1).

B) Barriers to discussing the discontinuation of cancer-directed therapies

Patient and family’s related barriers were also considered the most important, namely patients’ and families’ poor appreciation of prognosis or denial of likely survival duration as well as patients’ inflated expectation of the benefits from further cancer-directed therapy (Fig. 1).

Regarding the physicians’ related barriers, the most selected was the difficulty in estimating patient prognosis/length of survival, the uncertainty of the benefits of further active cancer therapy, and patient age (whereby had more difficulty suspending active treatment in younger patients) (Fig. 1).

The lack of guidelines for discontinuing cancer-directed therapy was also ranked as an important external barrier (Fig. 1).

III) Barriers to early palliative care referrals.

**Table 1:** Sociodemographic profile of the 66 oncologists in the sample

Variable	Descriptive
Age, median [1ºQ; 3ºQ], min–max	40 [36; 45], 28–68
Sex, n (%)	
Female	38 (57.6)
Male	28 (42.4)
Ethnicity, n (%)	
White	43 (65.2)
Black/Pardo	21 (31.8)
Asian	1 (1.5)
Other	1 (1.5)
Religion, n (%)	
Catholic	42 (63.6)
Agnostic	12 (18.2)
Evangelic	6 (9.1)
Spiritualism	5 (7.6)
Cristian	1 (1.5)
Years in oncology, median [1ºQ; 3ºQ], min–max	11.5 [8; 15], 2–39
Regions of medical service, n (%)	
Southeast	27 (40.9)
North	17 (25.7)
Northeast	10 (15.1)
Midwest	06 (9.1)
South	06 (9,1)
Work at palliative care services at some point, n (%)	
No	33 (50)
Yes	33 (50)

The most described barriers were lack of access to palliative care services, lack of allied health support team (social workers, nurse practitioners, etc.) to aid in the patient referral process to palliative care, and the patient or family refusal of referral (Fig. 1).

Most of the oncologists self-reported an average level of skills in goals of care communication (*n* = 34; 51.5%) and indicated learning communication skills as a high priority (*n* = 39; 59.1%). Nevertheless, most already had formal training regarding this topic (*n* = 38; 57.6%) and considered the quality of the training had moderately high (*n* = 19; 50%).

Comparing the two groups: participants who had formal training on communication about goals of care and those who did not have any training, some statistically significant differences were found. Patients’ lack of use of advanced directives (*p* = 0.007) was found to be a more significant barrier in discussing advance care planning and goals of care by the group with training on communication about goals of care, as well as the lack of decision aids and guidelines for discontinuing cancer-directed therapy (*p* = 0.023; *p* < 0.001). Participants with formal training ranked the lack of access to palliative care services within the hospital as a more important obstacle to referral to early palliative care (*p* = 0.012). Furthermore, they revealed more willingness to participate in decision-making about goals of care (*p* = 0.006) (Table 2).

**Table 2:** Barriers scoring differences according to the report on formal training regarding communication with patients and families about goals of care (*N* = 66)

BARRIERS	Formal training regarding communication with patients and families about goals of care		
	**No (*n* = 28)** Med [1^st^ Q; 3^rd^ Q]	**Yes (*n* = 38)** Med [1^st^ Q; 3^rd^ Q]	**Mann–Whitney’s *p*-value**
A) Barriers to Discuss ACP and goals of care			
Barriers Related to the Patient/Family			
Patient does not have an advance directive	6 [5; 6.5]	6 [6; 7]	0.014*
B) Barriers to Discontinuation of Cancer Therapies			
External barriers			
Lack of decision aids for discontinuing cancer-directed therapy	5 [3; 6]	6 [5; 6]	0.045*
Lack of guidelines for discontinuing cancer-directed therapy	4 [2; 6]	6 [5; 6]	< 0.001*
C) Timing of Palliative Care Referral			
Lack of access to palliative care services within the hospital	5 [3.5; 7]	6.5 [5; 7]	0.024*
D) Willingness to Participate in Communication and Decision-Making About Goals of Care			
Rate your willingness to initiate the discussion (bring up the subject) about goals of care with patients such as these and their families	6 [6; 7]	7 [6; 7]	0.006*
Rate your willingness to lead the discussion with patients such as these and their families. This includes exchanging information (disclosing diagnosis, prognosis, and eliciting values) and being a decision coach (clarifying values, assisting with weighing options for care, etc.)	6 [6; 7]	7 [6; 7]	0.013*

^*^ *p* < 0.05, *Med* Median, *Q* Quartile

When comparing the group of participants who have already worked in palliative care services with those who have not, the group with experience in palliative care also considered the lack of patients’ advanced directives (*p* = 0.007) as an important barrier to goals of care discussion and stated more willingness to participate on decision-making about goals of care (*p* = 0.001; *p* = 0.01). Particularly, the group with palliative care experience was perceived as a significant barrier to patients’ difficulty in understanding their prognosis/length of survival (*p* = 0.018) and patients’ inflated expectation of the benefits from further cancer-directed therapy (*p* = 0.004), as well as the lack of sufficient allied health support (social workers, nurse practitioners, etc.) to aid in patient support/referral process to palliative care (*p* = 0.012), compared to the group of participants without experience in palliative care (Table 3).

**Table 3:** Barriers scoring differences according to the report of palliative care experience (work at palliative care services at some point) regarding communication with patients and families about goals of care (*N* = 66)

BARRIERS	Work at palliative care services at some point		
	**No (*n* = 33)** Med [1^st^ Q; 3^rd^ Q]	**Yes (*n* = 33)** Med [1^st^ Q; 3^rd^ Q]	**Mann–Whitney’s *p*-value**
A) Barriers to Discuss ACP and goals of care			
Barriers Related to the Patient/Family			
Patient does not have an advance directive	6 [ 5; 6]	7 [ 6; 7]	0.014*
Patients’ difficulty understanding their prognosis/length of survival	6 [ 6; 7]	7 [ 6; 7]	0.037*
B) Barriers to Discontinuation of Cancer-Directed Therapies			
Patient and family barriers’			
Patient’s inflated expectation of the benefits from further cancer-directed therapy	6 [ 5; 6]	6 [ 6; 7]	0.008*
C) Timing of Palliative Care Referral			
Lack of sufficient allied health support (social workers, nurse practitioners, etc.) to aid in the patient support/referral process	6 [ 5; 6]	6 [ 5; 7]	0.023*
D) Willingness to Participate in Communication and Decision-Making About Goals of Care			
Rate your willingness to initiate the discussion (bring up the subject) about goals of care with patients such as these and their families	6 [ 6; 7]	7 [ 6; 7]	0.003*
Rate your willingness to lead the discussion with patients such as these and their families. This includes exchanging information (disclosing diagnosis, prognosis, and eliciting values) and being a decision coach (clarifying values, assisting with weighing options for care, etc.)	6 [ 6; 7]	7 [ 6; 7]	0.02*

^*^ *p* < 0.05, *Med* Median, *Q* Quartile

### Qualitative analysis

#### Strategies and suggestions to improve decision-making about goals of care in clinical practice

Two categories of responses were identified: Intrinsic factors (related to the physician) and extrinsic factors (related to the health system) (Table 4).

**Table 4:** Strategies and suggestions to improve decision-making about goals of care in clinical practice (*N* = 39)

Intrinsic factors (Strategies related to the doctor)	n (%)
Early conversation about prognosis	15 (38.5)
Emotional aspects	11 (28.2)
Education in communication skills and palliative care	11 (28.2)
A good physician–patient relationship	08 (20.5)
Others	06 (15.3)
Extrinsic factors (Strategies Related to the system)	n (%)
Access to palliative care and interdisciplinary teams	10 (25.6)
Availability to discuss goals of care	08 (20.5)

A total of 39 participants (59%) answered the open-ended question about the strategies and suggestions to improve decision-making about goals of care in their clinical practice. Intrinsic factors that could positively impact in goals of care discussion, such as the capacity to talk about the prognostic earlier in the course of the disease, were one of the most described strategies (*n* = 15; 38.5%), like this participant cited: “*Initial explanation about diagnosis/prognosis and available therapeutic lines and outcome expectations”* (P8). This was followed by emotional aspects (*n* = 11; 28.2%), as described: *“Be honest and transparent with the patient and family, without taking away their hope*” (P48). Continuous education in communication skills and palliative care (*n* = 11; 28.2%) was also frequently described: *“Increased staff awareness of the importance of palliative care from the outset in the patient with metastatic disease” (P48),* “*Training on this since graduation and medical residency could facilitate this approach*” (P59). Various participants (*n* = 8; 20.5%) also describe that a good physician–patient relationship could help to improve communication and the decision-making process about goals of care, for instance: “*get to know the patient well, their values, their experiences. Have a good relationship with patient and family”* (P60) (Table 4).

Also, ten participants (25.6%) described extrinsic factors such as access to palliative care and interdisciplinary teams: “*Joint follow-up with the palliative care team earlier”* (P19, P49). Another extrinsic factor observed frequently was to have enough time to discuss goals of care (*n* = 8;20.5%): “*Have enough time for bonding, since low ability to understand patients and family members and a culture of denial of the finitude process, especially in young patients is common.*” (P36); “*Good relationship with patient and family and to have time for patient and family education*” (P46) (Additional file 1).

## Discussion

In this study, the barriers to goals of care discussion and ACP most perceived by Brazilian oncologists were those mainly related to patients’ and families’ factors, such as the difficulty for patients and their families to understand and accept their prognosis and lack of an advanced directive. These findings were similar to the results from two Canadian multicenter studies [12, 14] that applied the *Decide-Oncology* instrument with the aim to perceive these barriers from the perspectives of physicians who assist cancer patients and also found patients and families’ difficulty accepting their prognosis/length of survival as the main difficulties to start the conversation about goals of care. A systematic review of ACP from the patients’ perspective showed that some of them reported initial resistance to participating in an ACP conversation because they fear being confronted with the life-threatening nature of their disease. Despite the initial resistance, most of the patients who completed the conversation were satisfied [18].

The results showed that physician-related barriers were the least frequently described as important. It is necessary to reflect on the perception of physicians about the most important barriers being patient-related, such as the difficulty of patients to understand and accept their prognosis, which can be related, at least in part, to the physicians’ difficulty in predicting and/or in communicating this prognosis in an assertive and clear way [2, 14, 19] since these physicians’ difficulties were also frequently described by oncologists in this study. Evidence also shows that feeling discomfort with goals of care discussion, fear of affecting patients’ hope and emotional coping and lack of training in communication strategies about end-of-life are some frequent difficulties reported by physicians, as well as lack of time to have the conversations [2, 11, 19, 20], which were also found in this study.

Regarding the discontinuation of directed cancer therapies, the patients’ barriers were also perceived as the most important by oncologists, in accordance with the findings of the Canadian studies [12, 14]. The desire of patients and their families to maintain all kinds of therapies, even those considered futile, could be related to the notion that ongoing cancer treatment is often connected with hope [12, 21]. This may reflect gaps in patient knowledge regarding the limitations and potential harms of cancer therapies at the end of life, as well as deficiencies in physicians’ ability to effectively communicate about prognostics [14]. It is known that early follow-up by palliative care could improve the communication with patients with advanced diseases in helping them to prepare for the end of life through shared decision-making among patients, their families and healthcare providers [22].

The lack of palliative care and multidisciplinary teams aligned with the care of cancer patients are common difficulties perceived by the participants who had formal training on goals of care and who had worked at palliative care services. The insufficient health support (social workers, nurse practitioners, psychologists, etc.) integrating teams to aid in the patient referral process to palliative care is still a common reality in hospitals, especially those without specialised palliative care services, which hinders these patients’ access to the necessary integrated care [23]. Also, the lack of patients’ advanced directives was one of the main barriers denoted by the aforementioned groups. In Brazil, there are still low rates of advanced directive records, and it is still very little discussed by healthcare professionals since many are unaware of this instrument and that it could facilitate the decision-making process about goals of care at the end of life [24, 25, 26, 27]. Professionals who work in palliative care teams tend to perform more advance directives with their patients, and therefore, they may be more sensitive to the importance of this instrument for decision-making and the impact of the lack of patient advance directives. A systematic review shows that structured communication tools may increase the frequency of discussions about and completion of advanced directives and the consistency between the care desired and the care being provided to patients with advanced disease [28]. Training health professionals through communication skills courses is a promising approach to changing communication behaviour and attitudes [29]. Continuous education about this issue from medical graduation until the course of medical practice is likely to improve this scenario [5, 29, 30].

In this study, the oncologists were asked about strategies that, in their experience, could facilitate the discussion with patients about goals of care. The intrinsic factors were the most underlined in this qualitative analysis, like to know how to talk about prognostics earlier to deal with emotional aspects while discussing goals of care and ACP with their patients, and having continuous education in communication skills and palliative care. Granek et al. (31), in a Canadian qualitative study about communication in end of life, found some similar oncologists’ strategies, such: being open and honest with patient; having ongoing, early conversations; communicating about modifying treatment goals; and balancing hope and reality. Schulman-Green et al. [19] explored American oncologists’ perceptions about goals of care communication in a qualitative study and described as important facilitators for the discussion of goals of care the oncologist’s practice experience and a supportive practice environment. In our study, the oncologists reported a median of 11.5 years in practice. Furthermore, most participants had received training on goals of care communication, which had a significant positive impact on the willingness to participate in the decision-making process about goals of care, compared with those who did not have this training, even though most of the participants did not consider their level of training high on this topic, which could contribute to the perception of the difficulties even among those with training.

There is a lack of studies that address barriers to discussing goals of care and ACP, especially in the Brazilian reality. Given the current challenges society is facing, such as the growing ageing population and the increase in chronic diseases, the implementation of ACP and the discussion of goals of care are imperative for humanised care [7, 19, 23]. This study allows understanding of the difficulties perceived by the oncologists in discussing ACP and goals of care with their patients from both a quantitative and qualitative perspective, enabling the future development of strategies to surpass them.

Limitations of this study include a small sample – there was a reduced response rate. Given that data collection occurred during the pandemic period by a coronavirus, most healthcare professionals were mentally and physically overwhelmed and overburdened. Further issue that may have contributed to the lower response rate was the vulnerable nature of this topic. This may be related to a selection bias, since not everyone feels comfortable talking about this issue. Another limitation was that not all participants answered the open questions considered for qualitative analysis. This may reflect the difficulty/interest of the participants in describing strategies to minimize barriers to discussing goals of care. Nevertheless, this study comprises oncologists from all regions of Brazil, which allow us to have an overview of the national reality. Moreover, differently from the previous studies, the results have been further analyzed according to the presence of any formal communication training or working experience at the palliative care, instead of comparing only according to years of medical experience.

## Conclusion

Barriers to the engagement of oncologists with patients with advanced cancer in Advance Care Planning and goals of care conversations were assigned specially to patients’ and families’ related difficulties. Nevertheless, physician-related difficulties, such as estimating patient prognosis/length of survival, constraints in the clinical practice environment, lack of time and lack of access/support for referral to palliative care, were also described. The continuous education regarding goals of care communication has been suggested as a strategy that can minimise these barriers. Although some findings apparently reflect structural issues, such as the lack of a well-integrated palliative care structure in the Brazilian health system, some barriers also considered important should be overcome with continuous education about communication in end-of-life care.

The present study also elucidates the importance of recognising different characteristics and values of the patient and family and highlights the necessity of adapting the training of oncologists to correctly identify the indicators and approaches to initiating goals of care and advance care planning discussion. Moreover, the identification of barriers that limit the discussion of ACP and early palliative care referrals could help to prioritise the next steps for future studies aimed at improving advance care planning, helping clinicians to better support patients and families through shared decision-making based on the patient’s values.

## Supplementary Information

**Additional file 1.**

## Data Availability

All data generated or analysed during this study are included in this published article.
