# Supplementary material for: Advance care planning and goals of care discussion: the perspectives of Brazilian oncologists
Source: BMC Palliat Care. 2022 Sep 22;21:165. doi: 10.1186/s12904-022-01052-w (PMC9502602; doi:10.1186/s12904-022-01052-w)
Supplement: Supplementary file 1 — Additional file 1. [file 12904_2022_1052_MOESM1_ESM.docx]

**SUPPLEMENTARY APPENDIX**

**Appendix 1. Suggestions to improve decision-making about goals of care in clinical practice (Open-ending questions)**

**Table S1- Strategies Related to the doctor (Intrinsic factors)**

| 1.1- Early conversation about prognosis |
| --- |
| *P3- “The longer the follow-up time with the patient, the easier it is to discuss end-of-life care”* |
| *P4- “Clarifications about this care at the beginning of the doctor-patient relationship”* |
| *P7- “Involve palliative doctors, patients and family members in the matter from the beginning of palliative treatment.”* |
| *P8- “Initial explanation about diagnosis/prognosis and available therapeutic lines and outcome expectations”* |
| *P11- “To clarify the prognosis from the first medical appointment”* |
| *P15- “The family must be well informed about the disease and prognosis from the beginning”* |
| *P18-“Start the conversation as early as possible; to discuss the therapeutic options since the diagnoses of metastatic scenario and show what can be done if treatment fails and what the evolution would be like if the disease progresses”* |
| *P23- “Explain from the beginning, when possible, the prognosis and possible evolution of the disease.”* |
| *P24- “Dialogue from the first medical appointment”* |
| *P27- “Explain from the beginning whether possible, the prognosis and possible evolution of the disease.”* |
| *P35- “Talking about it from the first medical appointment”* |
| *P36- “Greater interaction between physician, multidisciplinary team and family”* |
| *P38- “Talk in advance with the patient and family about the goals of care and refer as soon as possible to the palliative care outpatient clinic.”* |
| *P46- “Early open and compassionate communication about goals that can be achieved with treatment.”*  *P60- “Clear conversations about prognosis, right from the start.”* |
| **1.2- Emotional aspects** |
| *P4- “A physician-doctor Relationship based on trust”*  *P5- “Empathy”*  *P6- “Humility”*  *P7- “Empathy with clarity in the way of communicating disease progression, limitation of therapeutic options...”*  *P19- “Speak with Honesty”*  *P24- “Trust and dialogue”*  *P35- “The proximity in the relationship with my patients”*  *P38- “Be honest with patients and family members. Do not create false hopes. I don’t like to talk about healing when the patient is already metastatic. I talk about disease and symptoms’ control.”*  *P41- “An empathic communication and a good doctor-patient relationship”*  *P48- “Be honest and transparent with patients and family members, without taking away their hope.”*  *P66- “Sincerity and clarity in the relationship with the patient”* |
| **1.3 Education in communication skills and palliative care** |
| *P5-* ***“****Training and technical evaluation”*  *P32- “Continuing education in palliative care and training of communication skills (to attend courses)”*  *P33- “Comprise the meaning of palliative care, explain the difference in palliative and terminal care”*  *P41- “Continuing Education in Communication, especially at the end of life”*  *P42- “Interdisciplinary teamwork and having training on communication skills”*  *P43- “Communication training”*  *P47- “Continuing education on breaking-bad news”*  *P48- ““Increased staff awareness of the importance of palliative care from the outset in the patient with metastatic.”*  *P56- “Training on Communication Skills; Continuing Education”*  *P59- “Empathic communication. Having training on this since graduation and medical residency could facilitate this approach”*  *P67- “Continuing education in communication skills, especially at the end of life.”* |
| **1.4- A good physician-patient relationship** |
| *P33- “Have a Good doctor-patient and family relationship”*  *P42- “Have a good doctor-patient relationship”*  *P58- “To Have Time for these conversations and good doctor-patient relationship”*  *P59- “A good doctor-patient relationship, good communication (clear, empathetic and assertive)”*  *P60- “Get to know the patient well, their values, their experiences. Have a good relationship with patient and family”*  *P61- “Knowing the patient well; good doctor-patient relationship. Have a multidisciplinary team (with psychologist working together)*  *P65- “Close doctor-patient relationship”*  *P67- “Good doctor-patient relationship, empathic communication”*  1.5- Others:  *P20- “Have an Advance directives”*  *P6- “Try to use words that are typical of the patient’s faith”*  *P20- “Clarity in the treatment objective, even if it is only palliative.”*  *P23 - “Improve communication with family and patient.”*  *P27- “Try to improve communication with family and patient.”*  *P58- “Give More information to the patient and family”* |

| Table S2- Strategies Related to the system (Extrinsic factors) |
| --- |
| 2.1- Access to palliative care and interdisciplinary teams |
| *P19- “Early access to Palliative Care approach”* |
| *P22- “To institute differentiated fees for exclusive palliative care and a better patients’ access to a multidisciplinary team”* |
| *P25- “Multidisciplinary meeting”* |
| *P26- “The main barrier is the lack of these services, especially in Brazil’s health care public system, which needs to be systematically and organised in oncology services. Only exist on paper. So, it is urgent and necessary to increase the offer of PC services in this system.”* |
| *P32- “Working in an interdisciplinary team”* |
| *P34- “Institution of official palliative care service and protocols in the healthcare system”* |
| *P43- “Have a palliative care team available”* |
| *P47- “An Interdisciplinary team”* |
| *P49- “Joint follow-up with the palliative care team earlier”* |
| *P57- “To have more time and a multidisciplinary team for these conversations”* |
|  |
| 2.2- Availability to discuss goals of care |
| *P8- “Have time for proper interaction with patient and family”* |
| *P26- “Often the family is the main barrier, as they do not want the patient to know the prognosis”* |
| *P34- “Time for the development of the doctor-patient relationship”* |
| *P35- “Have enough time for interaction with the multidisciplinary team and family”* |
| *P36- “Have enough time for bonding, since low ability to understand patients and family members and a culture of denial of the finitude process, especially in young patients is common.”* |
| *P46- “Have a good relationship with the patient and time for patient and family education”* |
| *P57- “To have enough time and a multidisciplinary team for these conversations”* |
| *P61- “To have more time and training for this kind of conversation”* |
